# Evolution of injury burden in Qatari professional football — 8 season data from the Aspetar Injury and Illness Surveillance Programme

**DOI:** 10.5114/biolsport.2025.139089

**Published:** 2024-08-08

**Authors:** Karim Chamari, Raouf Nader Rekik, Mokhtar Chaabane, Souhail Chebbi, Ramadan Daoud, Cristiano Eirale, Yorck Olaf Schumacher, Montassar Tabben, Roald Bahr

**Affiliations:** 1 Aspetar Orthopedic and Sports Medicine Hospital, Doha, Qatar; 2 Oslo Sports Trauma Research Centre, Norwegian School of Sport Sciences, Oslo, Norway

**Keywords:** Qatar, Epidemiology, Soccer, Injury Prevention, Preventive measure

## Abstract

Prospectively collected injury surveillance data are essential for designing and implementing injury prevention programmes. We investigated the incidence, characteristics and patterns of professional football injuries in Qatar, providing details on the most observed injuries’ burden. We prospectively recorded individual time-loss injuries and training/match exposure from 17 professional football teams in Qatar during 8 seasons (2014/15 to 2021/22). Injury definitions and data collection procedures followed the 2006 consensus statement and results reported according to the 2020 IOC consensus statement on football injuries and methodology of epidemiological studies on injuries, respectively. In total, 1466 players with 4789 registered injuries were followed. The overall injury burden was 129 [95% CI: 128–130] days/1000 h. Over the 8 seasons there was a significant decreasing trend in the incidence of gradual onset injuries (p = 0.0012) and a non-significant decreasing trend for suddenonset match injuries (p = 0.063). The injury burden for match injuries was greater than the burden resulting from training injuries (460 [95% CI: 460–460] vs 56 [95% CI: 55–57] days/1000 h, p < 0.0001). There was no difference in time loss between index and recurrent injuries. Hamstring muscle strain represented the most frequent injury with a median of 11 (inter-quartile 5–20) days to return to play (RTP). ACL complete tear was the most impactful injury, in term of return to play, with a median of 200 (116–253) days to RTP. Re-injuries constituted 10.8% (4.7% of exacerbations). Mean illness incidence was 1.1 (SD = 0.4) illness/1000 hours, representing 5 illnesses per squad per season, with no variation over time. Qatari professional football is characterized by an overall injury pattern and risk similar to Asian and European norms. There was a significant decreasing trend in the incidence of gradual onset injuries and a non-significant decreasing trend for sudden-onset match injuries.

## INTRODUCTION

In professional football, injuries not only affect player health but also have a significant impact on team success [1], in addition to the financial cost for the club. Prospectively collected injury surveillance data are essential for designing and implementing injury prevention programmes. To assess the effectiveness of any prevention plan implemented, a long-term trend analysis of the injury incidence, burden and pattern is needed.

To date, many epidemiological studies have investigated injuries in professional male footballers [1–7]. However, except for the UEFA studies, based on the largest European dataset in injury epidemiology, very few studies have had sufficient data allowing a proper longitudinal analysis [8–10]. Regional differences in injury characteristics have been reported, e.g. between Europe and Asia [11, 12]. The few available epidemiological studies in Asian professional football are either based on a single team or a few teams followed over a limited number of seasons [4, 5, 7, 11, 13–15], which limits the ability to assess evolution over time.

Some recent studies have focused on injury burden as an important variable to guide clinicians in their management of injury rehabilitation and return-to-play [16]. In this context, the Aspetar Sports Injury and Illness Prevention Programme aims at implementing injury prevention strategies within the professional league in Qatar, the Qatar Stars League (QSL) by (i) setting up knowledge translation mechanisms to share scientific research results [17], and (ii) allowing each football team to develop risk management plans and compare their experience against benchmark data from the league. Therefore, the aims of this study were to: (1) investigate the injury rates and patterns during 8 seasons at the professional football club level in Qatar; (2) provide details about the injury severity and burden of the most commonly observed injuries.

## MATERIALS AND METHODS

### Study design

This is an observational cohort study collecting injury data using the Sport Medicine Diagnostic Coding System (SMDCS) to classify injuries [18].

### Setting and participants

We prospectively recorded individual time-loss injuries and training/match exposure in adult (≥ 18 years old) male professional footballers from Qatar through 8 seasons (July 2014 to May 2022, inclusive). Seventeen teams (12 first and 5 second division league) were followed throughout the domestic season, as well as periods of international camps or tournaments. We included teams that provided at least six consecutive months of data and fulfilled the minimum standard for data quality. We included players who were either a first-team squad member or training regularly with the first team. Players with pre-existing injuries at the start of each season were included in the study only after successful return to play from these conditions. Players newly recruited to a club were included from their recruitment date.

The team physician in each club was in charge of collecting the data, using standardized tools. We distributed a detailed study manual outlining the details of data collection to the contact person before the team’s enrolment in the study. We also organized demonstration sessions every time a new team physician joined the programme. We recorded data using a custom-made Microsoft Office Excel file for quick data entry. Injury cards were also provided in Microsoft Office Word to assist clinicians in taking notes during daily clinical activity, prior to entry into the master data file. We asked the clubs to submit their data every month by email. According to the IRB approved research protocol (ADLQ-IRB: E2017000252), team physicians or physiotherapists verbally informed all players about the purposes and procedures of the study and the latter provided verbal consent before being included in the study.

### Data sources

To facilitate comparison with previous studies, injury definitions and data collection procedures followed the 2006 consensus statement on epidemiological studies in football and the IOC model for recording illnesses. [19, 20] The results are reported according to the 2020 IOC consensus statement on injury and illness epidemiology. [20]

We recorded all injuries resulting in a player being unable to fully participate in training or match play (i.e. time-loss injuries). The player was considered injured until the team medical staff allowed full participation in training and availability for match selection. We also reported individual player exposure during training sessions and matches (individual session duration for each player, in min). We did not record injuries that did not cause time off from football activities, or injuries occurring outside football activities.

### Variables

We recorded the following characteristics for each injury: diagnosis, onset (sudden vs. gradual), severity (number of days of time loss), injury: (i) type, (ii) body part, and (iii) mechanism (sprinting, tackling, twisting/change of direction, kicking, jumping, stretching and other), (iv) index injury (first injury of any type recorded during the study), exacerbation, or re-injury (injury to the same body part and same structure type within one year from index injury), (v) training or match injury (cases of gradual-onset injuries, where the injury could not be clearly attributed to a specific session, were classified as not applicable). We classified cases as missing data when, despite all efforts to retrieve the data, the information could not be obtained. For the illnesses, diagnosis, number of days of time loss, and affected system (from a predefined list) were recorded. We classified cases as missing data when, despite all efforts to retrieve the data, the information could not be obtained.

We classified injury severity according to the duration of time loss as follows: mild (0–3 days), minor (4–7), moderate (7–28) and severe (> 28). [19]

### Illness

#### Data management

Prior to July 2018, diagnosis was recorded as free text written by the team doctors. From July 2018 on, the SMDCS classification system was introduced to the data collection tools. All the previous diagnoses were converted to SMDCS classification separately by two team doctors. Mismatched diagnoses were then discussed by the same two doctors for agreement. When agreement could not be reached, the diagnosis was reported as “unsure” and considered as missing information during the data analysis.

#### Statistical analyses

There were no statistically significant differences in injury incidence (p = 0.78) and burden (p = 0.20) between the division 1 and division 2 leagues. We therefore merged the data from both divisions. We present descriptive data as the mean with standard deviations or 95% confidence intervals (CI) unless otherwise noted. We used the median and interquartile range (IQR) to report injury severity. We calculated the injury incidence as the number of time-loss injuries per 1,000 player hours and the injury burden as the total number of days lost per 1,000 player hours of exposure. We used a simple linear regression to analyse the trend of injury incidence over seasons.

## RESULTS

### Population and exposure

In total, 1,466 players were followed up during the 8 seasons. The characteristics of teams, exposure and players over the 8-season observation period are presented in Table 1.

**TABLE 1:** Player population and exposure characteristics.

Season	2014/15	2015/16	2016/17	2017/18	2018/19	2019/20	2020/21	2021/22	Total
Teams (n)	16	14	15	17	17	17	17	17	130

Team-months (n)	155	134	144	180	179	165	179	166	1 302

Total exposure (h)	100 260	67 115	86 304	104 336	97 881	86 086	107 676	96 726	746 384

–Training	89 880	58 739	76 326	92 835	87 140	76 735	92 533	83 010	657 198 (88.1%)

–Match	10 381	8 376	9 977	11 501	10 741	9 351	15 142	13 716	89 186 (11.9%)

Player-seasons (n)	425	410	486	477	484	593	490	425	3 365

Players per team (n)	30 ± 4.5	31.1 ± 4.5	27.9 ± 4.3	29.2 ± 6.8	29.5 ± 5.6	29.4 ± 5.9	41.2 ± 12.1	30 ± 4	31.2 ± 7.8

Age (years)	26.4 ± 4.7	26.6 ± 4.4	26.5 ± 4.6	26.5 ± 4.5	26.3 ± 4.6	26.5 ± 5.1	26.3 ± 5	26.3 ± 5.3	25.6 ± 5

Body mass (kg)	70.5 ± 6.8	74 ± 8.2	72.2 ± 8.9	74.9 ± 9.4	74.4 ± 9.8	73.9 ± 8.9	73.4 ± 9.6	73.4 ± 8.8	72.7 ± 9.4

Height (cm)	168.9 ± 19.1	173.8 ± 17.3	174.7 ± 14.9	167 ± 32.6	166.9 ± 31.9	176.3 ± 12	176.7 ± 10.3	177.4 ± 7.5	174.2 ± 19.3

The mean overall exposure per player to football during one full season was 162 ± 92 h, consisting of 142 ± 82 h of training and 21 ± 15 h of match play. The average training-to-match ratio across the eight seasons was 7.5 ± 1.0.

### Injury incidence

In total, 4,789 injuries were registered. Of these, 3,924 (81.9%) were reported as sudden onset (41.6% during match, 51% during training and 7.4% as missing information) and 808 as gradual onset (16.9%), while for 57 injuries (1.2%) information was missing.

The overall injury incidence was 6.4 [95% CI: 6.3 to 6.6] injuries/1,000 h, with greater incidence during matches (19.3 [95% CI: 17.5 to 19.3]) injuries/1,000 h) compared to training (3.1 [95% CI: 2.9 to 3.2] injuries/1,000 h) (p < 0.0001).

**FIG. 1:**
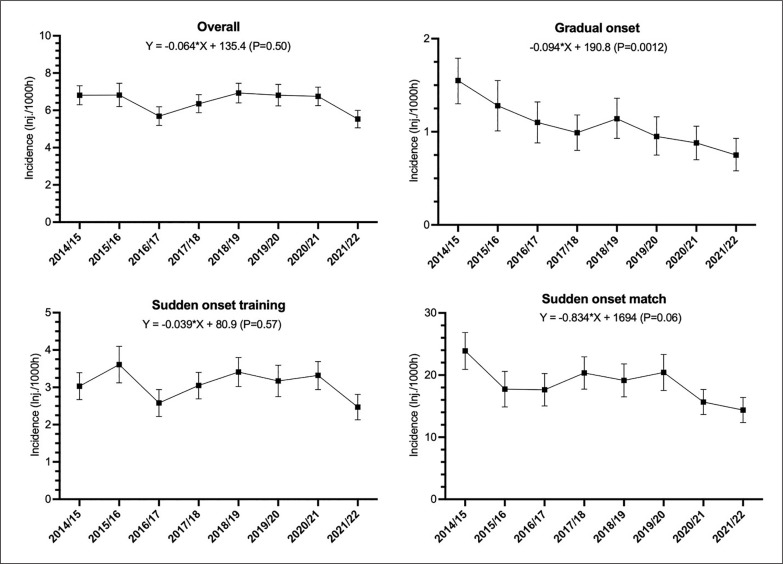
Incidence of overall injuries, sudden onset injuries during matches and training, and gradual-onset injuries (number of injuries per 1,000 h with 95% confidence intervals as error bars) over the 8 seasons.

The incidence of overall, sudden onset match and training, and gradual onset injury over 8 seasons is presented in Figure 1. There was a significant decreasing trend in the incidence of total gradual onset injuries (p = 0.016) and a non-significant decreasing tendency for match sudden-onset injuries (p = 0.063).

### Injury burden and severity

The overall injury burden was 129 [95% CI: 128 to 130] days/1,000 h. The injury burden for match injuries was greater than the burden resulting from training injuries (460 [95% CI: 460 to 460] vs 56 [95% CI: 55 to 57] days/1,000 h, p < 0.0001). The majority of injuries were moderate (n = 2,093; 43.2%) followed by minor (n = 1,166; 24.1%), severe (n = 819; 16.9%) and mild (n = 711; 14.7%); 54 cases (1.1) had not returned to play at the end of the study or had missing return to play dates.

**FIG. 2:**
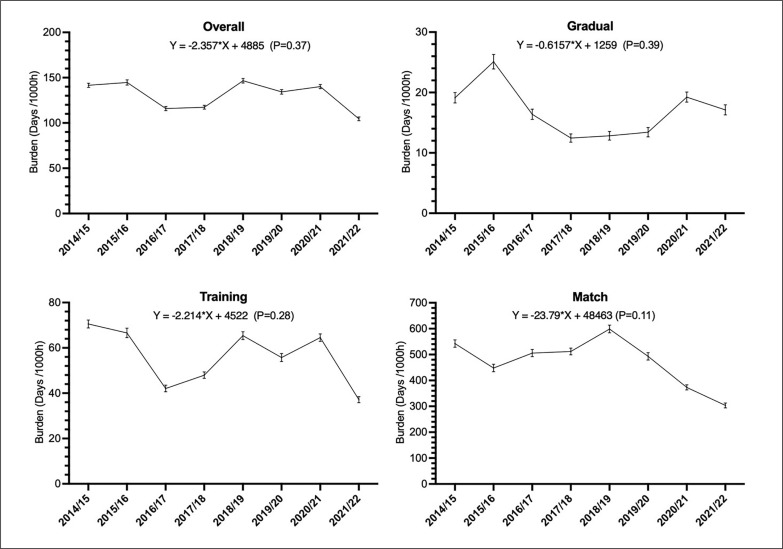
Injury burden over the 8 seasons for (i) overall, (ii) gradual-onset injuries, and (iii) training and (iv) match sudden-onset injuries (days lost per 1,000 h of exposure with 95% confidence intervals as error bars).

The injury burden of overall, sudden-onset injuries that occurred during matches or training sessions, and gradual-onset injuries over the 8 seasons are presented in Figure 2. There was no significant trend of variation over the 8 seasons regarding all injuries’ burden.

**FIG. 3:**
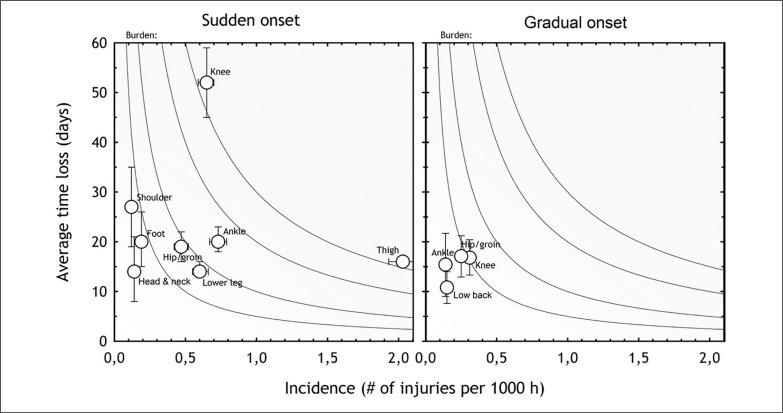
Risk matrix depicting the relationship between incidence (number of injuries per 1,000 h) and severity (average number of days lost) with 95% confidence interval for each body region that were split into sudden and gradual onset mechanism of injury. The darker the colour, the greater the burden; isobars depict a burden of 5, 10, 20 and 30 days per 1,000 h respectively (from bottom to top lines). Only body regions with an injury burden (average number of days lost per 1,000 h) of > 1 and a minimum number of > 3 cases are included in the figure.

Figure 3 represents a risk matrix based on the incidence and severity of the most commonly reported injured body parts, separately by mode of onset.

Thigh and knee injuries represented the greatest injury incidence and burden, respectively (Table 2). The two specific injuries causing the greatest burden were complete ACL ruptures (0.1 injuries per team per month, resulting in a median of 200 days lost) and hamstring strains (0.6 injuries per team per month resulting in a median of 12 days lost).

**TABLE 2:** Injury pattern by body region, injury type, and diagnosis.

	No. of Injuries	Severity Median (IQR) days	Incidence Injuries/1,000 h (95% CI)	Burden Days lost/1,000 h (95% CI)
Head & neck	105	6 (2 to 12)	0.14 (0.11 to 0.16)	1.6 (1.6 to 1.7)
Facial fracture	18	15 (11 to 28)	0.02 (0.01 to 0.04)	0.5 (0.5 to 0.6)
Concussion	23	8 (5 to 12)	0.03 (0.02 to 0.04)	0.3 (0.3 to 0.3)

Shoulder	86	15 (7 to 31)	0.12 (0.09 to 0.14)	3.2 (3.1 to 3.3)
Dislocation	17	29 (11 to 44)	0.02 (0.01 to 0.03)	1.1 (1.1 to 1.2)

Elbow & arm	21	13 (4 to 37)	0.03 (0.02 to 0.04)	0.6 (0.5 to 0.6)

Hand	68	8 (3 to 18)	0.09 (0.07 to 0.11)	1.3 (1.2 to 1.3)

Low back	199	4 (2 to 9)	0.27 (0.23 to 0.3)	2.4 (2.3 to 2.5)

Hip & groin	491	9 (4 to 18)	0.66 (0.6 to 0.71)	12.1 (11.8 to 12.3)
Proximal adductor strain	272	10 (5 to 20)	0.36 (0.32 to 0.41)	6.8 (6.7 to 7)

Thigh	1 356	11 (5 to 20)	1.82 (1.72 to 1.91)	27.5 (27.1 to 27.8)
Hamstring strain	761	12 (6 to 20)	1.02 (0.95 to 1.09)	15.8 (15.5 to 16)
Quadriceps strain	266	15 (5 to 25)	0.36 (0.31 to 0.4)	7 (6.8 to 7.2)
Adductor strain	210	9 (4 to 16)	0.28 (0.24 to 0.32)	3.5 (3.3 to 3.6)

Knee	610	12 (5 to 38)	0.81 (0.75 to 0.88)	34.7 (34.2 to 35.1)
Cartilage/synovium/bursa	225	14 (5 to 30)	0.3 (0.26 to 0.34)	7 (6.8 to 7.2)
Lateral meniscal tear	38	21 (12 to 52)	0.05 (0.03 to 0.07)	2 (1.9 to 2.1)
Medial meniscal tear	42	29 (12 to 48)	0.06 (0.04 to 0.07)	2 (1.9 to 2.1)
Osteoarthritis	92	9 (4 to 18)	0.12 (0.1 to 0.15)	1.8 (1.7 to 1.9)
Ligament/joint capsule	249	27 (8 to 119)	0.33 (0.29 to 0.37)	25.7 (25.3 to 26)
ACL complete tear	80	200 (116 to 253)	0.11 (0.08 to 0.13)	20.4 (20.1 to 20.7)
MCL sprain	120	12 (5 to 31)	0.16 (0.13 to 0.19)	3.2 (3 to 3.3)

Lower leg	416	9 (3 to 19)	0.56 (0.5 to 0.61)	8.3 (8.1 to 8.6)
Muscle strain	311	11 (5 to 20)	0.42 (0.37 to 0.46)	6.3 (6.1 to 6.5)
Bone	28	29 (4 to 55)	0.04 (0.02 to 0.05)	1.5 (1.4 to 1.6)
Fracture	17	50 (27 to 91)	0.02 (0.01 to 0.03)	1.3 (1.3 to 1.4)

Ankle	527	8 (4 to 20)	0.71 (0.65 to 0.77)	14.2 (14 to 14.5)
Ligament/joint capsule	364	11 (5 to 23)	0.49 (0.44 to 0.54)	9.8 (9.5 to 10)
Lateral ligament sprain	223	9 (4 to 19)	0.3 (0.26 to 0.34)	4.6 (4.4 to 4.8)
Syndesmosis injury	51	38 (22 to 61)	0.07 (0.05 to 0.09)	3.2 (3.1 to 3.3)
Deltoid ligament sprain	70	12 (4 to 19)	0.09 (0.07 to 0.12)	1.5 (1.4 to 1.6)
Fracture	10	91 (65 to 99)	0.01 (0.01 to 0.02)	1.1 (1.1 to 1.2)

Foot	137	5 (2 to 17)	0.18 (0.15 to 0.21)	3.6 (3.5 to 3.8)
Fracture	28	52 (16 to 87)	0.04 (0.02 to 0.05)	2.1 (2 to 2.2)

Other	49			

Missing information	9			

Apart from concussion and facial fracture, only subgroups with an injury burden of > 1.1 days lost/1,000 h are included in the table.

### Injury mechanisms

Sprinting was the most common injury mechanism for a total of 889 injuries (29%), followed by other (752; 24%), twisting/change of direction (581; 19%), kicking (474; 15%), tackling (283; 9%), stretching (241; 8%), and jumping (237; 8%), while for 524 injuries (17%) information was missing.

### Reinjuries

Reinjuries represented 10.8% and exacerbations 4.7% of all injuries, resulting in a mean time loss of 20.0 and 21.5 days, respectively. Overall, all reinjuries (including exacerbations) resulted in 20.1 days of time loss, with no significant trend of variation over the 8 seasons.

### Illnesses

The incidence for illness was 1.1 [95% CI: 0.9 to 1.3] per 1,000 h, corresponding to 5 illnesses per squad (25 players) per season, with no time trend during the 8 seasons (Y = -0.01874*X + 38.91, P = 0.8088). The most commonly affected systems were the upper respiratory tract (51%) followed by the gastrointestinal system (14%).

## DISCUSSION

The key novelty of the study is that it is the first to look at trends in one professional league. Our study is the first to follow the 2020 IOC and FIFA consensus statements by presenting the results of gradual- and sudden-onset injuries separately [20, 21]. Over the 8 seasons the overall injury risk was stable, while the incidence of gradualonset injuries declined. Hamstrings, quadriceps and ACL injuries remain concerning.

## Injury incidence and trend

The average 8-season injury incidence in the Qatari first league was 6.4 [95% CI: 6.3–6.6] injuries/1000 h. This is consistent with a previous study from a decade ago (6.0/1000 h) reporting overall injury data from the same league (Eirale et al., 2013a). However, the match injury rate was higher (19.3 vs 14.5/1000 h, respectively) while the rate during training was lower (3.1 vs 4.4/1000 h, respectively) than the previous study. The observed overall match and training injury rates were within the ranges reported from the UEFA Champions League teams [6, 22]. The data from our prospective study rely on solid methodology [17, 20, 21], with committed researchers handling the data management and data quality control [23], ensuring robust results.

During the 8 seasons we observed a significant decreasing trend in gradual-onset injuries and there was also a non-significant decline in match injuries. In contrast, Ekstrand et al. [6, 22] prospectively observed stable incidence of training and match injuries over seven and 11 seasons in Europe, respectively. The main trends longitudinally reported in the UEFA study concern decreases in (i) ligament injuries over seven seasons [24] and (ii) specific ankle sprains over eleven seasons [25], while (iii) hamstring muscle strains increased over thirteen seasons [26]. However, they did not report on sudden- and gradual-onset injuries separately. We are the first to report a significant decrease of gradual-onset injuries over time.

Our study design does not allow us to identify the reasons behind this decrease. Ekstrand et al. [26] commented on the role of medical staff in professional football, stating that clinicians are usually following the frontline of sports medicine knowledge with specific attention to injury prevention. In Qatari football this access to knowledge is systematic, as all the clinicians working with the professional football teams belong to the same institution (Aspetar) and participate in a consistent, continuing education programme with a particular focus on football medicine and injury prevention. Therefore, there is a possibility that this has had an impact on the observed reductions, even though team medical staff encounter several barriers when attempting to implement injury prevention programmes [27].

Despite the stable overall and training sudden-onset injury incidence rates, there was a non-significant decreasing trend of match sudden-onset injuries. This goes against the increase in hamstring injuries reported by Ekstrand et al. [26] over 13 years. The latter authors did not split the hamstring injuries into sudden- or gradual-onset injuries but rather into training and match injuries. For the match injuries, they reported a non-significant increase, while the training hamstring injuries significantly increased by 5% yearly. We can assume that most of the latter study match hamstring injuries were of sudden onset, although this was not mentioned by the authors.

Figure 3 shows that averaged monthly rates progressively increased to reach a peak in November for mean overall, training and match sudden-onset injury incidence. This period represents the end of the first half of the seasons before the mid-season break that usually takes place in December in Qatari professional football. The seasonal variation approach was analysed by Ekstrand et al. [6], showing that compared to the pre-season there was a higher risk of hamstring injuries during the competitive season, peaking in April for the end of the competitive season.

The risk matrix, which depicts the correlation between injury severity and incidence, can be a useful tool to guide risk management and determine injury prevention priorities [16]. Importantly, the risk matrix depicted in Figure 3 clearly shows that gradual-onset injuries did not represent the main concern for the QSL teams. Sudden-on-set injuries represented a much bigger problem, with a substantial injury burden caused by thigh (hamstring and quadriceps muscle injuries) and knee injuries (particularly ACL ruptures). These data should inform the priorities when developing prevention programmes. ACL tears resulted in a similar median time loss (200 days) as in previous research in Asia (197 days) and Europe (205 days) (Ekstrand et al., 2020; Tabben et al., 2022). However, our ACL injury incidence (0.11 injuries/1000 h) was slightly higher than in a previous study in Qatar (0.08 injuries/1000 h) [7] and the UEFA study (0.06 injuries/1000 h), [10] but lower than in the AFC study (0.14 injuries/1000 h). [11] These discrepancies could be related to the different conditions of play according to geographical locations, climatic conditions [28] and pitch characteristics (and/or player skills and muscular strength [12]. These observations call for further investigation to improve our understanding of injuries’ aetiology and mechanisms.

The overall injury burden was 129 days/1000 h. This result is higher than in the AFC study with 112 days/1000 h and similar to the UEFA study with 130 days/1000 h [11, 29]. The training injury burden in our study was higher than in the AFC, but lower than in the UEFA studies (56.0, 54.0 and 60.5 days/1000 h of exposure, respectively). For matches, it was similar to AFC and lower than UEFA (460.0, 456.0 and 504.6 days/1000 h, respectively). The most notable difference with European elite football was for matches. This could be explained by greater match exposure in Europe (average of 60 matches played by the first team per season) [30] than in Qatar (always less than 45 official matches per season).

## Reinjuries

Our reinjury proportion was similar to Asian and European football (10%, 9.9%, and 10–12%, respectively) [11, 29], while being lower than the previously reported Qatari data from a decade ago (15%) [7]. The severity of injury was the same between index injuries and reinjuries (21 and 20 days), again improving compared to the past data (21.4 vs 37.5 days, respectively) [1] and being lower compared to the AFC study (23 and 25 days, respectively). In the UEFA study, the reinjury rate decreased over time and only six diagnosis types had a significantly higher average time loss for the reinjury compared to the index injury [29]. The return to play decision is generally made by the club doctor, the coach and the player in a shared decision-making process which usually results in more cautious timeline for reinjury compared to the index injury, resulting in longer lay-off.

The low reinjury rate (nearly 10%) recently reached in Europe and Asia (including our study), and its decrease during the last years, could be explained by better medical support and risk management protocols now being used in professional football clubs.

Exacerbations (i.e. reinjury occurring before RTP) represented ~5% of all injuries (season range ~3 – ~8%). However, we were unable to compare it to the literature as it is still an underreported aspect of injury epidemiology.

## Illnesses

In our study the seasonal illness incidence ranged from 0.6 to 1.9 illnesses/1000 h (average 1.1 ± 0.4 illnesses/1000 h). This is a slightly higher rate than reported in previous research in football, like the AFC study reporting an incidence of 0.9 illnesses/1000 h [11], values that are also consistent with previous research in the national league in Norway [31]. Furthermore, consistent with previous research in Asia and Europe, the respiratory tract was the system most affected by time loss illnesses.

## Study limitations

The injuries’ time loss definition results in missing medical attention conditions (overuse injuries). These, though not stopping the player from training or competing, result in a substantial amount of pain/discomfort, as is the case for groin problems [32, 33]. The potential under-reporting of some clinicians, the non-optimal use of Excel files to manage data, and the difficult working conditions during the COVID-19 pandemic might have impacted the quality of our data.

## CONCLUSIONS

Qatari professional football is characterized by an overall injury pattern and risk similar to Asian and European norms. Over the 8 seasons, we observed a high incidence and burden of ACL ruptures, hamstring and quadriceps injuries. However, there was a significant decreasing trend in the incidence of gradual onset injuries and a non-significant decreasing trend in sudden-onset match injuries.

## Data sharing statement

The datasets generated and/or analysed during the current study are not publicly available due to being held securely in coded form, but are available in coded form from the corresponding author on reasonable request.

## Patient involvement

no
